# Palliative Debulking of an Aortic Body Tumour With Right Atrial Invasion in a Dog Under Partial Cardiopulmonary Bypass

**DOI:** 10.1002/vms3.71244

**Published:** 2026-09-23

**Authors:** Shuji Suzuki, Sachiyo Tanaka, Nobuo Kanno, Takuya Yogo, Yasuji Harada, Masaki Michishita, Yasushi Hara

**Affiliations:** ^1^ Laboratory of Veterinary Surgery Nippon Veterinary and Life Science University Musashino Tokyo Japan; ^2^ Laboratory of Veterinary Pathology Nippon Veterinary and Life Science University Musashino Tokyo Japan

**Keywords:** ascites, cardiac surgery, chemodectoma, extracorporeal circulation, intracardiac mass

## Abstract

**Background:**

Aortic body tumours are uncommon heart‐base neoplasms in dogs, and intracardiac extension causing clinically significant right atrial obstruction is rarely described.

**Case presentation:**

A 7‐year‐1‐month‐old, 15.2‐kg, castrated male French Bulldog was evaluated for 2 weeks of lethargy, hyporexia, exercise intolerance, abdominal distension, and polydipsia. Transthoracic echocardiography revealed severe right‐sided cardiac enlargement and a heterogeneous right atrial intracavitary mass (3.2 × 3.1 cm) suspected to arise from the heart base, with tricuspid regurgitation (maximal velocity 4.03 m/s). Abdominal ultrasonography showed hepatic venous congestion and marked ascites; fluid was a modified transudate. Medical therapy failed to resolve ascites, and surgical debulking was performed on Day 7 under partial cardiopulmonary bypass. The mass was firmly adherent to the atrial septal aspect and could not be completely excised. Postoperatively, ascites and clinical signs resolved, right‐sided cardiac enlargement decreased, and tricuspid regurgitation was no longer detectable; the dog was discharged on Day 14. A recurrent right atrial mass was documented on Day 360 without recurrence of right‐sided heart failure. The dog died on Day 390 from pancreatitis‐associated biliary obstruction and multiple organ failure. Necropsy confirmed an aortic body tumour with intracardiac extension into the right atrium and pulmonary metastasis with tumour emboli.

**Conclusion:**

Partial cardiopulmonary bypass‐assisted palliative debulking may temporarily relieve life‐limiting intracardiac obstruction in carefully selected dogs; however, this approach is high‐risk and non‐curative, and its contribution to long‐term survival remains uncertain because postoperative toceranib therapy and the natural behaviour of the tumour may also have influenced the clinical course.

## Graphical abstract

This report describes a French Bulldog that developed severe right‐sided congestive heart failure due to mechanical obstruction of venous return by a heart‐base tumor extending into the right atrium. Partial cardiopulmonary bypass‐assisted palliative debulking relieved the obstruction and temporarily improved clinical signs and ascites. This case highlights a potential role for surgical palliation when rapid relief of life‐limiting intracardiac obstruction is required.

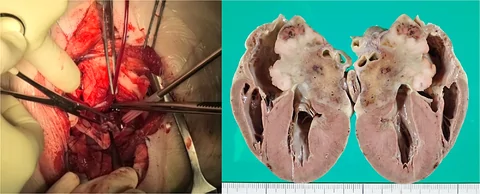

## 1 Introduction

Aortic body tumours (chemodectomas) are typically slow‐growing heart‐base neoplasms in dogs (Vicari et al. 2001; Yamamoto et al. 2013), and treatment options include medical management, radiation therapy, tyrosine kinase inhibitors (Coto et al. 2021; Magestro et al. 2018; Kruckman‐Gatesy et al. 2020; Hansen et al. 2021), pericardectomy when clinically indicated, and surgical intervention in selected cases. Although these tumours often cause clinical signs by compressing adjacent cardiovascular structures, intracavitary or intracardiac involvement is uncommon (Cho et al. 1998; Crawford‐Jennings et al. 2025). Those reports documented intracardiac involvement of canine aortic body tumours but did not describe tumour debulking under cardiopulmonary bypass (CPB). CPB has been used for selected canine intracardiac tumour procedures, including surgical treatment of a left atrial paraganglioma and a tricuspid valve myxoma (Buchanan et al. 1998; Kanno et al. 2025). However, reports specifically describing partial CPB‐assisted palliative debulking of a canine heart‐base tumour extending into the right atrial lumen are lacking. The objective of this case report was to describe the clinical presentation, diagnostic findings, partial cardiopulmonary bypass‐assisted palliative right atrial tumour debulking, and outcome in a dog with an aortic body tumour extending into the right atrium.

## 2 Case Presentation

A 7‐year‐1‐month‐old, 15.2‐kg, castrated male French Bulldog was referred for further evaluation and treatment of lethargy, hyporexia, exercise intolerance, and abdominal distension of approximately 2 weeks’ duration. Abdominal ultrasonography performed by the referring veterinarian revealed ascites, but no overt abdominal disease was identified as a primary cause of the effusion. Transthoracic echocardiography disclosed marked right‐sided cardiac enlargement and a right atrial mass, prompting referral.

**FIGURE 1:**
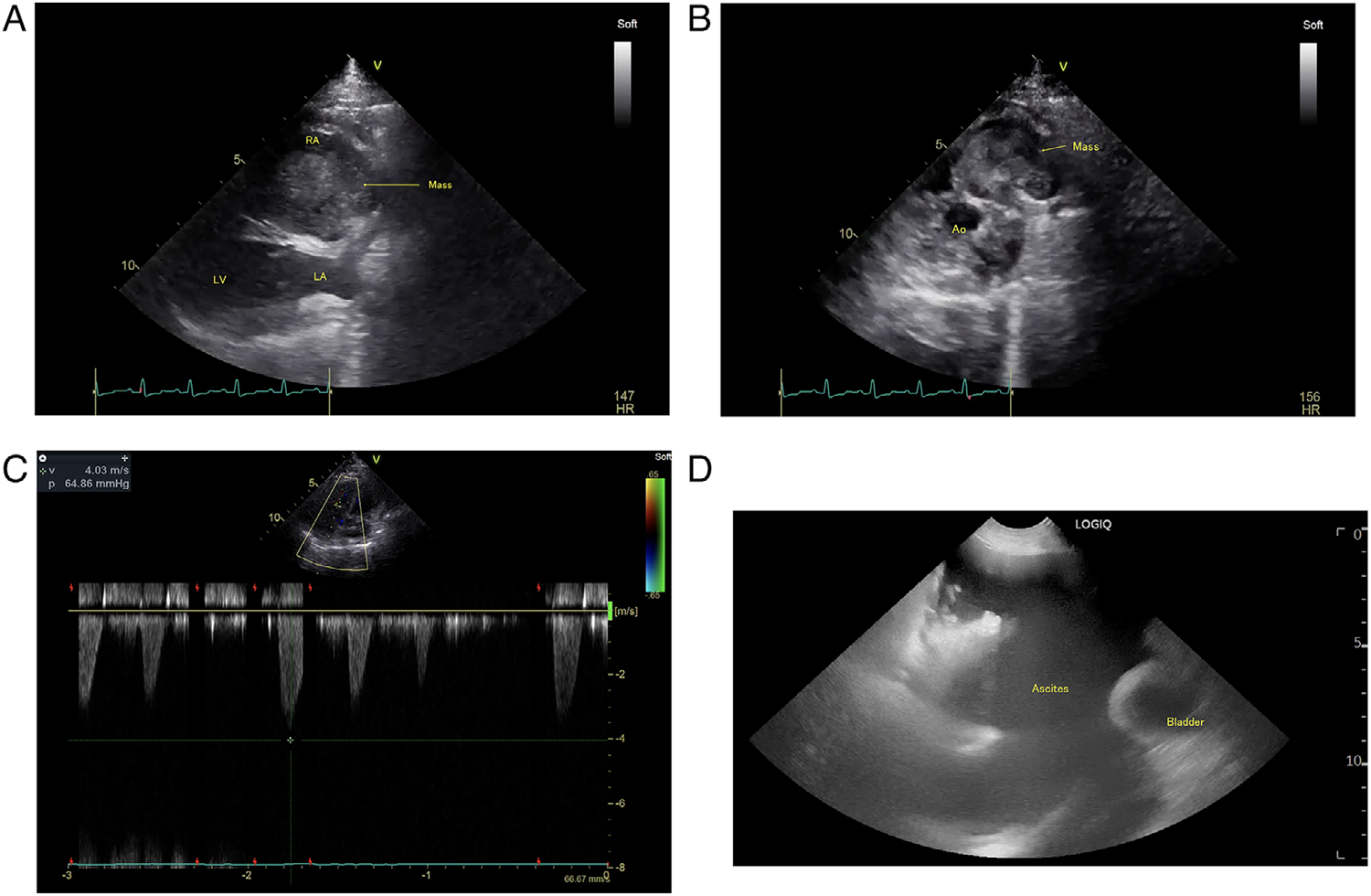
Echocardiographic and abdominal ultrasonographic findings at initial presentation. (A) Right parasternal long‐axis four‐chamber view showing marked right atrial enlargement and a large intracavitary mass adjacent to the interatrial septum. The mass was heterogeneously echogenic. (B) Right parasternal short‐axis view at the heart base demonstrating that the mass appeared to originate from the heart base region adjacent to the aorta; the maximal dimensions were 3.2 × 3.1 cm. (C) Continuous‐wave Doppler recording of tricuspid regurgitation obtained from a left parasternal long‐axis view. The peak tricuspid regurgitant velocity was 4.03 m/s (estimated pressure gradient, 64.9 mmHg). (D) Abdominal ultrasonography showing a large volume of anechoic free fluid cranial to the urinary bladder (ascites). Abbreviations: Ao, aorta; LA, left atrium; LV, left ventricle; RA, right atrium.

At initial presentation, body temperature was 38.0°C, heart rate was 147 bpm, and respiratory rate was 30 breaths/min. Mucous membranes were pale, and abdominal distension was evident. Indirect arterial blood pressure was 147/76 mmHg (mean, 110 mmHg). Electrocardiography showed sinus rhythm without arrhythmia. Blood typing identified the dog as DEA 1.1‐negative. Haematology revealed anaemia (PCV 30.3%; Table 1). FDP and D‐dimer concentrations were increased (Table 1). Serum biochemistry showed increased ALT activity and a mildly increased C‐reactive protein concentration; the remaining clinicopathological findings are summarised in Table 1. Echocardiography demonstrated severe right‐sided cardiac enlargement and an intracavitary mass (3.2 × 3.1 cm) extending into the right atrial lumen, adjacent to the interatrial septum, and originating from the heart base region (Figures 1A, 1B, Video S1). The mass was heterogeneously echogenic with an irregular surface. Tricuspid regurgitation was present with a maximal regurgitant velocity of 4.03 m/s (estimated pressure gradient, 64.9 mmHg) (Figure 1C). Paradoxical interventricular septal motion was observed. Abdominal ultrasonography revealed hepatic venous congestion and a large volume of ascites (Figure 1D). Thoracic radiographs obtained before surgery did not reveal radiographically apparent pulmonary metastases. Abdominal ultrasonography showed no obvious evidence of distant metastasis. Preoperative CT angiography and thoracic CT were not performed, and no additional abdominal staging procedures beyond ultrasonography were undertaken. Therapeutic abdominocentesis was performed to relieve abdominal distension, removing approximately 600 mL of fluid. Ascitic fluid had a specific gravity of 1.028 and was classified as a modified transudate. Based on these findings, ascites secondary to the right atrial mass was suspected (differential diagnoses included thrombus and neoplasia). Medical treatment was initiated with pimobendan (0.25 mg/kg PO twice daily), furosemide (1 mg/kg PO twice daily), and low‐molecular‐weight heparin (100 IU/kg SC twice daily). Despite therapy, ascites persisted, and the dog's activity and appetite remained decreased.

**TABLE 1:** Clinicopathological findings at presentation.

Parameter	Result	Unit	Reference interval
**Haematology and haemostasis**			
RBC	4.35	x10^6/µL	5.50‐8.50
Hb	10.4	g/dL	12.0‐18.0
PCV	30.3	%	37.0‐55.0
MCV	69.3	fL	60.0‐77.0
MCHC	34.3	g/dL	32.0‐36.0
Platelets	445	x10^3/µL	200‐500
WBC	8600	/µL	6000‐17,000
PT	8.9	sec	6.0‐9.5
APTT	13.1	sec	9.0‐16.5
Fibrinogen (FBG)	122	mg/dL	150‐350
Antithrombin III (ATIII)	164.5	%	70‐160
FDP	6.8	µg/mL	0.0‐4.0
D‐dimer	4.1	µg/mL	0.0‐2.0
**Serum biochemistry**			
Glucose	143	mg/dL	75‐128
Total protein	4.9	g/dL	4.9‐7.2
Albumin	2.4	g/dL	2.0‐3.2
ALT	310	U/L	14‐68
AST	45	U/L	14‐44
ALP	130	U/L	47‐254
BUN	11.9	mg/dL	9.2‐29.2
Creatinine	0.72	mg/dL	0.40‐1.45
CK	99	U/L	47‐168
Phosphorus (P)	4.7	mg/dL	1.9‐5.0
Calcium (Ca)	9.1	mg/dL	9.1‐12.3
Sodium (Na)	142	mmol/L	141‐152
Potassium (K)	4.9	mmol/L	3.8‐5.1
Chloride (Cl)	107	mmol/L	102‐117
CRP	1.58	mg/dL	0.0‐1.0
Total cholesterol (T‐Cho)	165	mg/dL	105‐322
Triglycerides (TG)	38	mg/dL	17‐113

Abbreviations: ALP, alkaline phosphatase; ALT, alanine aminotransferase; APTT, activated partial thromboplastin time; AST, aspartate aminotransferase; ATIII, antithrombin III; BUN, blood urea nitrogen; CK, creatine kinase; CRP, C‐reactive protein; FDP, fibrin/fibrinogen degradation products; Hb, haemoglobin; MCHC, mean corpuscular haemoglobin concentration; MCV, mean corpuscular volume; PCV, packed cell volume; PT, prothrombin time; RBC, red blood cell count; T‐Cho, total cholesterol; TG, triglycerides; WBC, white blood cell count.

The differential diagnoses for the right atrial mass included neoplasia, thrombus, and tumour‐associated thrombus. Thrombus or tumour‐associated thrombus could not be completely excluded preoperatively. However, the dog had received antithrombotic therapy from the referring veterinarian, and no clinical improvement or reduction in the size of the mass was observed. In addition, the imaging findings and clinical course were considered more consistent with a space‐occupying intracardiac lesion causing mechanical obstruction of the right atrium. Preoperative cytology or biopsy was not performed because the mass was located within the right atrium, and tissue sampling was considered technically difficult and potentially hazardous. The owner was informed that the tumour type had not been definitively diagnosed before surgery, that complete excision might not be possible, and that the procedure was intended to be palliative rather than curative. Non‐surgical and palliative options, including continued drainage and diuretic therapy, antithrombotic therapy, medical treatment such as toceranib, radiation therapy, pericardectomy, and euthanasia, were discussed with the owner. Pericardectomy was not expected to address the primary problem because the major lesion was an intracavitary right atrial mass causing mechanical obstruction of venous return, rather than pericardial effusion or constrictive pericardial disease. These non‐surgical or less invasive options were considered unlikely to rapidly relieve the mechanical obstruction of the right atrium. Because the predominant clinical problem was considered to be mechanical obstruction of venous return rather than functional myocardial failure alone, medical management alone was considered unlikely to provide rapid relief. The expected benefit of surgery was temporary relief of life‐limiting right atrial obstruction, improved venous return, and resolution of ascites, whereas the major risks included incomplete tumour removal, recurrence, fatal haemorrhage, cardiac injury, inadequate cardiopulmonary bypass, tumour embolisation or dissemination, and perioperative death. Therefore, surgical debulking of the right atrial mass under partial cardiopulmonary bypass (CPB) was selected as a rescue palliative intervention and was performed on Day 7. Before surgery, detailed informed consent was obtained from the owner. The owner was also informed that partial cardiopulmonary bypass might not be achievable or might be insufficient, and that severe complications, including fatal haemorrhage, cardiac wall or septal injury, ventricular fibrillation, failure to separate from cardiopulmonary bypass, postoperative thrombosis, tumour embolisation or dissemination, recurrence, and perioperative death, could occur.

Premedication consisted of midazolam (0.3 mg/kg IV), fentanyl (5 µg/kg IV) and cefazolin sodium (25 mg/kg IV). After preoxygenation (100% O_2_), anaesthesia was induced with propofol IV to effect and maintained with sevoflurane (1.5%–3%). The right jugular vein and right carotid artery were surgically exposed and isolated for cannulation. A 14‐Fr venous drainage cannula was placed in the right jugular vein and advanced through the cranial vena cava, with its tip positioned near the cranial vena cava–right atrial junction. An 8‐Fr arterial cannula was placed in the right carotid artery.

A right fifth intercostal thoracotomy was performed, the heart was exposed, and a pericardial tent was created. The azygos vein was not intentionally occluded or ligated. Although temporary azygos vein occlusion can reduce venous return into the operative field during cardiopulmonary bypass, the azygos vein was deliberately left unoccluded in this case because venous return from the caudal body was considered to be compromised by the right atrial mass and impaired caudal vena caval inflow. Preserving collateral venous return through the azygos system was considered preferable, even though it could contribute to some reduction in surgical visibility. Additional venous cannulation from the right atrium toward the caudal vena cava (16‐Fr) was attempted before tumour debulking but could not be advanced because of interference by the right atrial mass. Therefore, partial CPB was performed using the cervical cannulas alone. Direct caudal vena caval venotomy and cannulation were not performed because of the anticipated risk of haemorrhage and deterioration of surgical visibility.

Heparin sodium (200 U/kg IV) was administered to achieve an activated clotting time (ACT) > 400 s. Partial CPB was established using a standard extracorporeal circuit. After initiation of CPB, ACT increased to approximately 1000 s. Blood flow was set at 60–80 mL/kg/min. Cooling was performed, and the lowest recorded esophageal temperature was 28°C. Serial arterial blood gas analyses were performed during partial CPB. During bypass, mean arterial pressure ranged from 58 to 120 mmHg. Arterial blood gas analyses showed pH 7.28–7.50, PaO_2_ 195–416 mmHg, PaCO_2_ 38–55 mmHg, bicarbonate 22.7–29.4 mmol/L, base excess −1.9 to 4.0 mmol/L, and lactate concentration 1.0–2.5 mmol/L. Central venous pressure during bypass and calculated oxygen delivery indices were not recorded. The decision to proceed with right atriotomy under this limited bypass configuration was based on maintaining a pump flow of approximately 60–80 mL/kg/min, acceptable systemic arterial pressure, serial arterial blood gas findings, lactate concentrations, and adequate surgical field control using suction. A strict predefined numerical abort criterion was not established.

**FIGURE 2:**
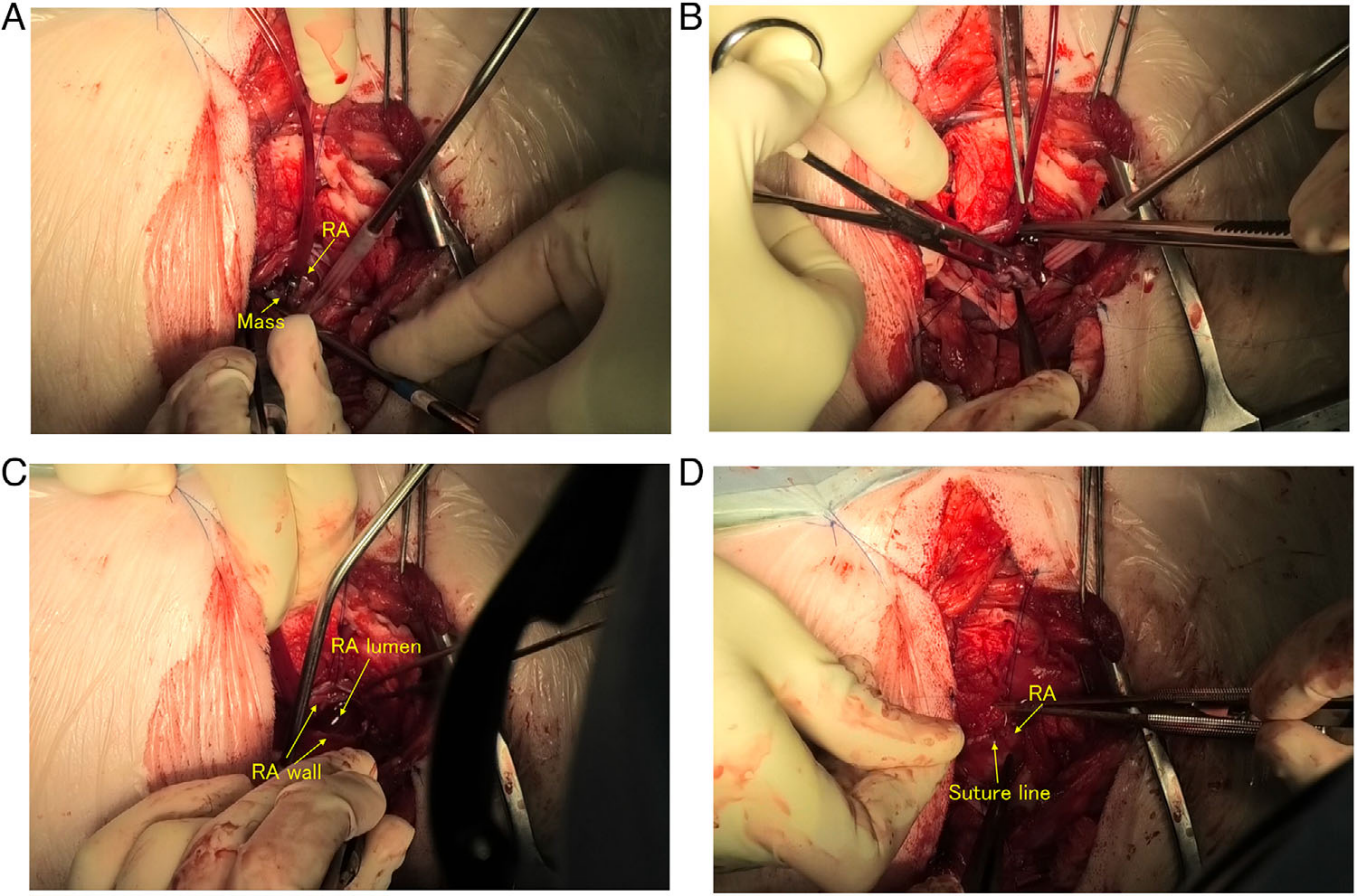
Intraoperative findings during debulking of the right atrial mass under partial cardiopulmonary bypass. (A) Intraoperative view immediately after right atriotomy showing that the mass occupied most of the right atrial lumen. (B) The intracavitary component of the mass was gently stabilised with Babcock forceps to improve exposure and was carefully debulked; excessive traction was avoided because the mass was firmly adherent to the septal aspect of the right atrium. (C) After debulking, blood flow from the caudal vena cava into the right atrium appeared improved, and the right atrial lumen refilled with blood. (D) The right atrium was closed in two layers with a simple continuous pattern after limited palliative debulking. The residual tumour was intentionally left at the firmly adherent portion to avoid rupture, perforation, or uncontrollable haemorrhage. Visible labels indicate the mass, right atrial wall, right atrial lumen, Babcock forceps, and suture line. Abbreviation: RA, right atrium.

The right atrium was incised, and the mass was found to occupy most of the right atrial lumen and to be firmly adherent to the septal aspect of the right atrium with marked induration, suggesting neoplastic tissue (Figure 2A, Video S2). Babcock forceps were used to gently grasp and stabilise the intracavitary component of the mass to improve exposure, rather than to forcibly avulse the tumour from the atrial wall. Debulking was performed primarily by blunt dissection with gentle traction, and sharp dissection was carefully used near the tumour attachment site to avoid inadvertent injury to the atrial or septal structures. A distinct dissection plane between the tumour and the right atrial wall was not clearly identifiable in the firmly adherent portion. Because the mass was markedly indurated and broadly attached to the septal aspect of the right atrium, complete separation from the atrial wall was not attempted. The residual tumour was intentionally left in place where further dissection was considered likely to risk perforation, rupture, uncontrollable haemorrhage, or injury to critical structures. No substantial haemorrhage from the attachment site or resected surface was observed, and no uncontrollable bleeding occurred. The aim of the procedure was palliative restoration of right atrial luminal patency rather than complete tumour excision.

After partial tumour debulking, venous return from the caudal vena cava into the right atrium increased, and caudal vena caval cannulation through the right atriotomy was reconsidered. However, venous return was managed using two suction lines, including one suction tip placed near the caudal vena caval orifice and an additional field suction line. Suctioned blood was returned to the extracorporeal circuit, and the drainage obtained with suction was considered sufficient to maintain the operative field. Therefore, additional caudal vena cava cannulation was not pursued, and the caudal vena cava remained uncannulated throughout the procedure. In collaboration with the perfusionist, suction and pump flow were adjusted to balance surgical visibility and systemic perfusion during right atrial tumour debulking.

The accessible intracavitary component of the mass was debulked to the extent considered technically safe until satisfactory restoration of right atrial luminal patency was achieved (Figures 2B, 2C). The right atriotomy was closed in two layers with a simple continuous pattern using 6‐0 polypropylene (Figure 2D). Approximately 10 min after atriotomy, ventricular fibrillation occurred; defibrillation was performed after completion of closure and resulted in return of sinus rhythm. Packed red blood cells (50 mL) and fresh frozen plasma (50 mL) were administered intraoperatively, and 50 mL of autologous blood collected preoperatively was transfused postoperatively. Total anaesthesia time was 410 min, total surgical time 350 min, total CPB time 140 min, and intracardiac manipulation time, defined as the time from atriotomy to completion of closure, was 40 min. The thorax was closed routinely.

Central venous pressure was measured intermittently using a 5‐Fr multipurpose catheter advanced into the cranial vena cava immediately proximal to the right atrium before placement of the venous drainage cannula and after completion of intracardiac manipulation and removal of the venous drainage cannula. Continuous central venous pressure monitoring was not performed during partial cardiopulmonary bypass. The available recorded values decreased from approximately 19 mmHg before tumour debulking to approximately 5 mmHg after debulking. The corresponding systemic arterial pressures were 104/63 mmHg (mean, 77 mmHg) and 118/81 mmHg (mean, 97 mmHg), respectively. Although caudal vena caval or hepatic venous pressure was not directly measured, the decrease in central venous pressure, together with postoperative resolution of ascites and improvement in echocardiographic findings, was considered consistent with improved venous return following partial relief of the right atrial obstruction.

Because suctioned blood was returned to the extracorporeal circuit, the theoretical risk of tumour‐cell recirculation was considered. The extracorporeal circuit and oxygenator were equipped with standard filters intended to remove air bubbles, thrombi, and fat droplets; however, these filters were not designed to reliably remove individual tumour cells or microscopic tumour fragments. Grossly visible tumour fragments were not identified in the operative field or suction reservoir, and the mass did not fragment or become detached when gently grasped and retracted, indicating that it was firm rather than friable. Nevertheless, microscopic tumour‐cell shedding or microscopic tumour‐fragment aspiration during manipulation and suction could not be excluded.

**FIGURE 3:**
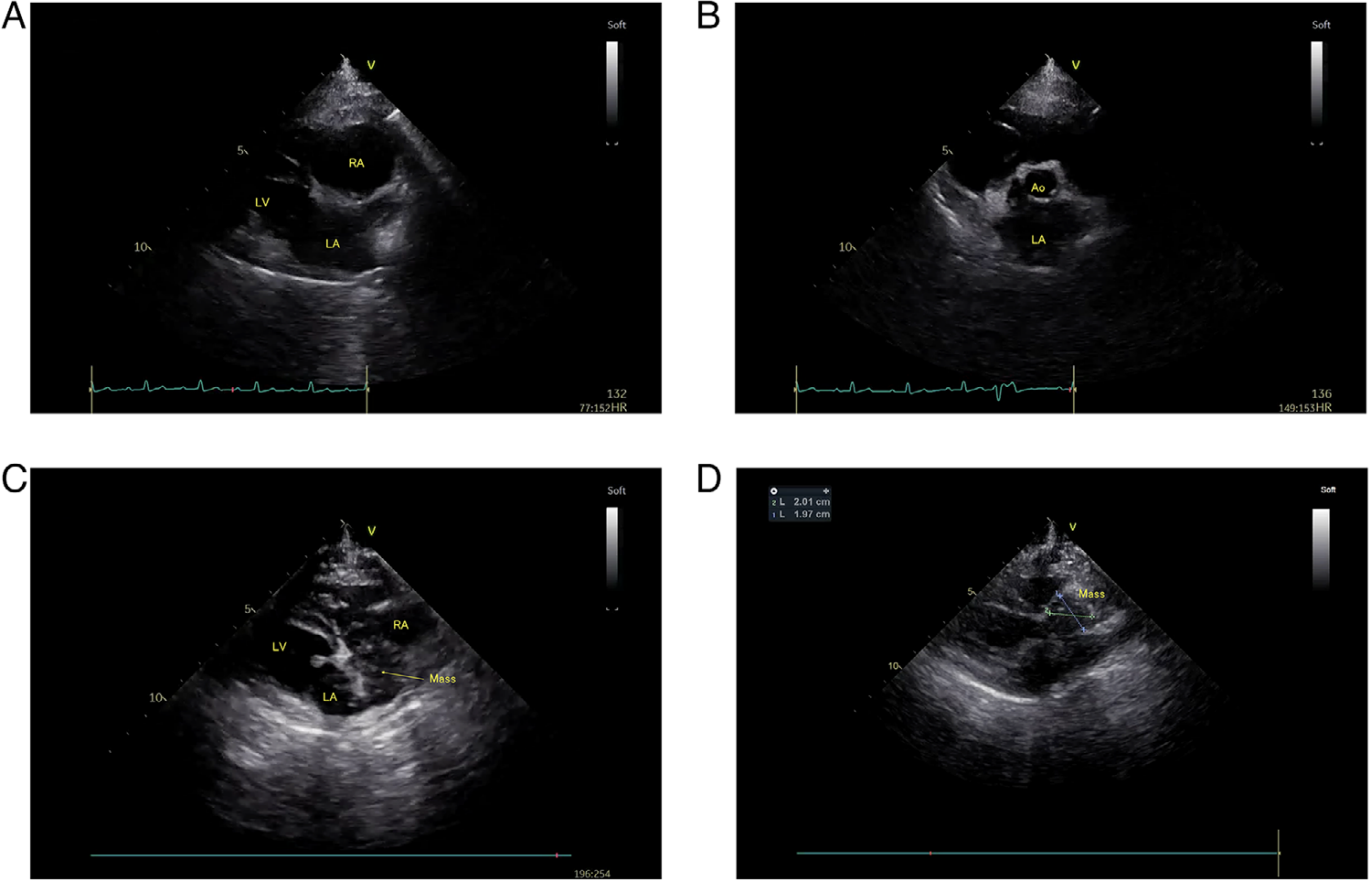
Postoperative echocardiographic findings. (A) Right parasternal long‐axis four‐chamber view on Day 14 showing that the intracavitary mass was no longer clearly visible. (B) Right parasternal short‐axis view at the heart base on Day 14. Similar to panel A, the mass was not clearly identified. (C) Right parasternal long‐axis four‐chamber view on Day 360 showing a documented recurrent mass adjacent to the interatrial septum. (D) Measurement of the recurrent mass, with maximal dimensions of 2.0 × 1.9 cm.

Postoperatively, clinical recovery was uncomplicated; mucous membrane pallor resolved, ascites resolved, and appetite and activity returned. Pimobendan and furosemide were discontinued. Clopidogrel (2 mg/kg PO once daily) was prescribed for approximately 1 month. Follow‐up echocardiography showed only minimal residual intracardiac tissue, a reduction in right‐sided cardiac enlargement, and the absence of tricuspid regurgitation (Figures 3A, 3B, Video S3). The dog was discharged on Day 14. The postoperative course and long‐term follow‐up are summarised in Table 2.

**TABLE 2:** Timeline of clinical events.

Day	Event	Key findings or treatment
Day 0	Initial presentation	Lethargy, hyporexia, exercise intolerance, abdominal distension, marked ascites and a right atrial intracavitary mass were identified.
Day 7	Surgery	Rescue palliative debulking of the right atrial mass was performed under partial cardiopulmonary bypass.
Day 14	Discharge	Ascites and clinical signs had resolved; right‐sided enlargement decreased; tricuspid regurgitation was no longer detected.
Day 21 onward	Postoperative medical therapy	Toceranib was initiated on Day 21, corresponding to 14 days after surgery, at 2.5 mg/kg PO every other day and administered as postoperative medical therapy.
Follow‐up period	Echocardiographic monitoring	Postoperative follow‐up was performed primarily by clinical assessment and echocardiography; no postoperative CT examination was performed. Only minimal residual intracardiac tissue was observed initially, and no obvious enlargement causing recurrent right‐sided congestive heart failure was identified before Day 360.
Day 360	Recurrence documented	A recurrent right atrial mass measuring 2.0 × 1.9 cm was documented without recurrence of right‐sided heart failure; Day 360 was defined as the time point at which recurrence was documented.
Day 390	Death and necropsy	The dog died from pancreatitis‐associated biliary obstruction and multiple organ failure; necropsy confirmed an aortic body tumour with right atrial extension and pulmonary metastasis with tumour emboli.

**FIGURE 4:**
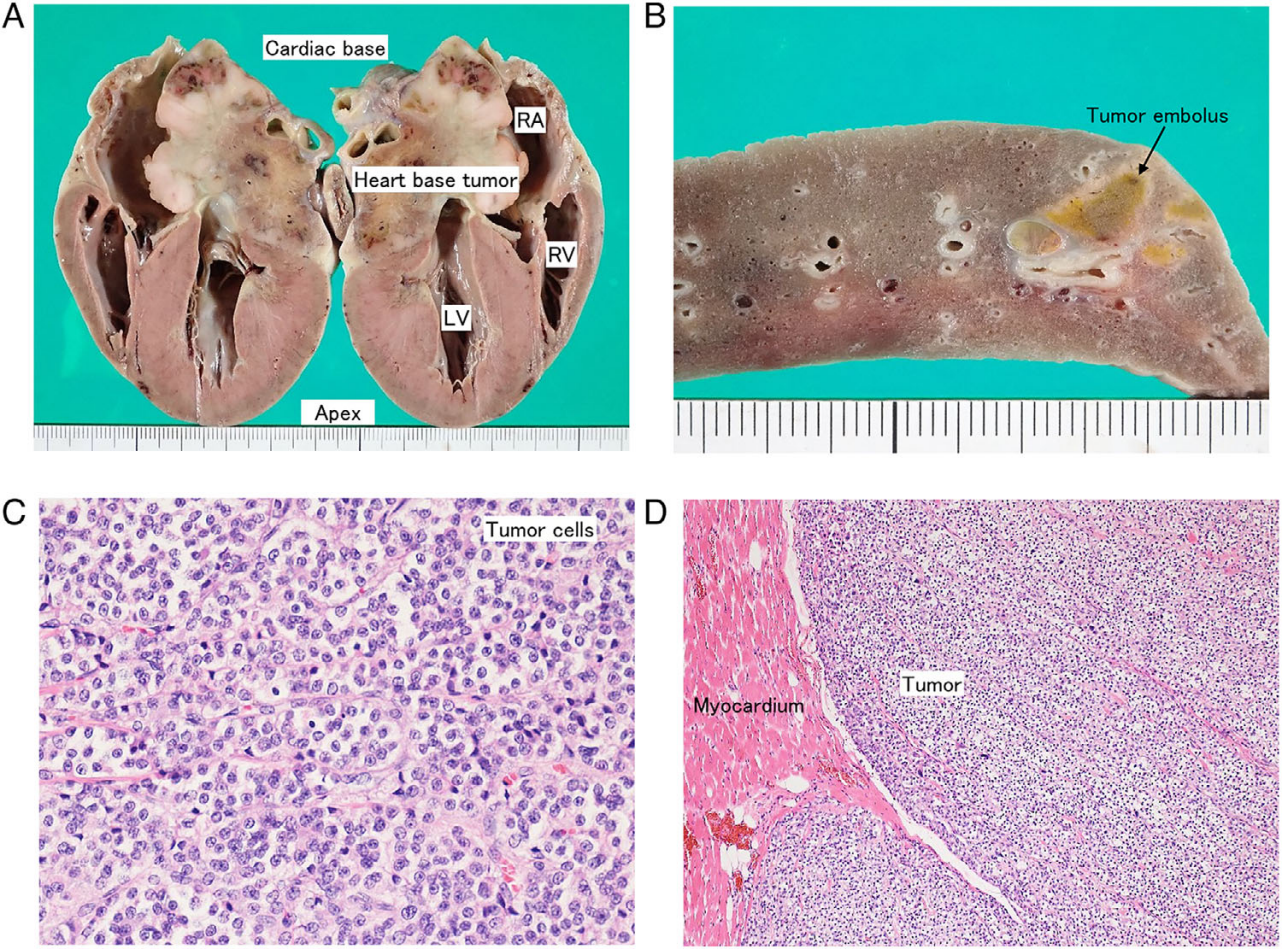
Gross and histopathological findings at necropsy. (A) Gross cross‐sectional image of the heart at necropsy showing the heart base tumour extending into the right atrium. The cardiac base and apex are indicated for orientation. (B) Cut surface of the left caudal lung lobe showing an intraluminal lesion within a pulmonary artery, consistent with thrombus/tumour embolus, and metastatic foci. (C) Histopathology of the cardiac mass: neoplastic cells resembling chemoreceptor cells proliferating in small‐ to medium‐sized nests separated by fibrovascular stroma (zellballen pattern) with areas of solid, sheet‐like growth (haematoxylin and eosin [H&E] stain; objective × 40). (D) At the cranial aspect of the left ventricular wall, the tumour infiltrated the myocardium (H&E stain; objective × 40). Abbreviations: LA, left atrium; LV, left ventricle; RA, right atrium.

Histopathology of the resected mass initially suggested a neuroendocrine neoplasm. Immunohistochemistry was positive for synaptophysin and chromogranin A and negative for thyroid transcription factor‐1 and cytokeratin AE1/AE3, supporting neuroendocrine differentiation. Toceranib (2.5 mg/kg PO every other day) was initiated on Day 21, corresponding to 14 days after surgery, as postoperative medical therapy. Postoperative follow‐up was performed primarily by clinical assessment and echocardiography; no postoperative CT examination was performed. No obvious intracardiac mass enlargement causing recurrent right‐sided congestive heart failure was identified during follow‐up before Day 360. On Day 360, a recurrent right atrial mass (2.0 × 1.9 cm) was documented without clinical signs of right‐sided heart failure (Figures 3C, 3D, Video S4). Day 360 was therefore considered the time point at which recurrence was documented; however, the true onset of tumour regrowth could not be determined precisely because regrowth was likely gradual and standardised follow‐up imaging at fixed postoperative intervals was not performed. On Day 390, the dog developed pancreatitis with biliary obstruction (total bilirubin, 12.8 mg/dL) and underwent computed tomography at a referral hospital (Video S5). Despite treatment, the dog died of multiple organ failure. Necropsy revealed a heart base mass with extension into the right atrium; the lesion infiltrated from the endocardial surface into the right atrial myocardium and protruded into the right atrial lumen. Pulmonary lesions were identified in the left caudal lung lobe. Histopathology at necropsy confirmed an aortic body tumour, and the pulmonary lesions were consistent with metastases and tumour emboli (Figures 4A, 4B, 4C and 4D).

## 3 Discussion

The literature regarding intracardiac extension and surgical treatment of canine aortic body tumours should be interpreted carefully. Cho et al. (1998) and Crawford‐Jennings et al. (2025) described intracavitary or intracardiac involvement of canine aortic body tumours, but these reports did not describe tumour debulking under CPB. Therefore, these reports support the rarity and potential invasive or metastatic behaviour of aortic body tumours, but they should not be interpreted as precedents for the surgical technique used in the present case.

Debulking or incomplete surgical management of chemodectoma and other cardiac tumours has been described in selected veterinary cases, but the techniques and indications differ substantially. More broadly, surgical management of canine heart‐base tumours has been described mainly as pericardectomy, surgical biopsy, or surgical excision in selected cases, and these approaches differ from intracardiac palliative debulking under CPB. For example, marginal surgical excision of a feline caval chemodectoma with vascular reconstruction and postoperative toceranib was reported, but that case involved the cranial vena cava rather than intracavitary right atrial obstruction and did not involve CPB (Martinez et al. 2022). In dogs, CPB has been used for selected intracardiac tumour procedures, including surgical treatment of a left atrial paraganglioma and a tricuspid valve myxoma (Buchanan et al. 1998; Kanno et al. 2025). However, to the authors’ knowledge, previous English‐language reports specifically describing partial CPB‐assisted palliative debulking of an aortic body tumour extending into the right atrial lumen in a dog could not be identified.

In this dog, an intracavitary mass adjacent to the interatrial septum occupied most of the right atrial lumen and was associated with marked ascites. The clinical presentation was most consistent with impaired venous return—particularly reduced inflow from the caudal vena cava—resulting in elevated right atrial pressure, hepatic venous congestion, and subsequent ascites. Following debulking of the intracardiac mass under partial cardiopulmonary bypass (CPB), ascites and associated clinical signs resolved, and the dog remained clinically stable without recurrence of right‐sided congestive heart failure until a recurrent right atrial mass was documented on Day 360. Necropsy ultimately confirmed an aortic body tumour with intramyocardial invasion and protrusion into the right atrial lumen. These findings suggest that, in selected dogs in which a heart base tumour extends intracardially and causes clinically significant obstruction, surgical debulking aimed at improving haemodynamics may provide temporary clinical palliative benefit.

Other potential causes of right‐sided heart failure or ascites were also considered. No overt primary abdominal disease was identified as the cause of the effusion, electrocardiography showed sinus rhythm without arrhythmia, and medical treatment, including diuretic and antithrombotic therapy, did not resolve the ascites. The postoperative resolution of ascites and clinical signs, reduction in right‐sided cardiac enlargement, disappearance of tricuspid regurgitation, and decrease in intermittently measured central venous pressure from approximately 19 mmHg before debulking to approximately 5 mmHg after debulking, without a simultaneous decrease in systemic arterial pressure, further supported mechanical obstruction by the right atrial mass as the main contributor to right‐sided congestive heart failure. Nevertheless, other contributing factors could not be completely excluded, and caudal vena caval or hepatic venous pressure was not directly measured.

The differential diagnosis for a right atrial mass includes thrombus, primary or metastatic neoplasia such as hemangiosarcoma, and infective vegetations (Fruchter et al. 1992; Karn et al. 2023). In the present case, the mass was broadly attached near the interatrial septal aspect and showed a hyperechoic/heterogeneous appearance, and its location at the heart base was suspected on echocardiographic views. Taken together with the necropsy findings, these features were compatible with an aortic body tumour extending into the right atrium. Although aortic body tumours most commonly arise at the heart base and are often locally expansive, metastatic disease and/or invasive growth have been reported in a subset of cases (Yamamoto et al. 2013; Johannsen et al. 2025). Intracardiac extension across the atrial septal region appears uncommon, and such extension can lead to severe right‐sided congestive heart failure due to mechanical obstruction of venous return and impaired right atrial filling. In this dog, the large‐volume ascites (a modified transudate) was considered secondary to increased right atrial pressure and hepatic venous congestion.

A definitive histologic diagnosis was not obtained before surgery. Therefore, at the time of surgical decision‐making, the lesion was an undiagnosed right atrial intracavitary mass, and the differential diagnoses included chemodectoma, hemangiosarcoma, ectopic thyroid carcinoma, thrombus, lymphoma, metastatic carcinoma, and other neoplastic or thrombotic lesions. These conditions have different prognostic and therapeutic implications, and the lack of preoperative tissue diagnosis represents an important limitation of this case. A less invasive biopsy approach was considered; however, tissue sampling of the intracardiac mass was considered technically difficult and potentially hazardous because the mass was located within the right atrial lumen, was associated with obstruction of venous return, and was adjacent to critical cardiac structures. Biopsy or manipulation before establishing surgical control was considered to carry risks of haemorrhage, cardiac wall injury, arrhythmia and tumour or thrombus embolisation. Therefore, the decision to proceed directly to surgery was based on the need for rescue palliative relief from life‐limiting right atrial obstruction rather than on a definitive oncologic diagnosis.

Medical management did not provide adequate clinical improvement, likely because the predominant pathophysiology was mechanical obstruction rather than functional myocardial failure alone. Other treatment modalities were also considered. Toceranib has been reported to provide clinical benefit in dogs with chemodectoma (Coto et al. 2021), and recent reports of conventional fractionated radiation therapy and stereotactic body radiation therapy for canine heart‐base tumours have described tumour volume reduction, clinical improvement or disease stabilisation (Magestro et al. 2018; Kruckman‐Gatesy et al. 2020; Hansen et al. 2021). Therefore, radiation therapy should be considered an important option, particularly for dogs with nonresectable or slowly progressive heart‐base tumours when immediate mechanical decompression is not required. In the present case, however, severe ascites, hepatic venous congestion, and a large intracavitary right atrial mass suggested life‐limiting mechanical obstruction of venous return, and radiation therapy was not expected to provide immediate relief of this obstruction. Thus, debulking of the right atrial mass under partial CPB was selected as rescue palliative treatment to improve venous return rather than as definitive oncologic therapy.

The bypass strategy in this case was limited because complete caudal vena caval cannulation was not achieved. Additional venous cannulation toward the caudal vena cava was attempted before tumour debulking, but the cannula could not be advanced because of interference by the right atrial mass. After partial tumour debulking, venous return from the caudal vena cava into the right atrium increased, and caudal vena caval cannulation through the right atriotomy was reconsidered. However, venous return from the caudal vena cava could be adequately managed using two suction lines, including one suction tip placed near the caudal vena caval orifice and an additional field suction line, and suctioned blood was returned to the extracorporeal circuit. Because the drainage obtained with suction was considered sufficient to maintain the operative field, additional caudal vena caval cannulation was not pursued in order to avoid prolonging intracardiac manipulation and total operative time during this limited palliative procedure. The caudal vena cava therefore remained uncannulated throughout the procedure. The azygos vein was deliberately left unoccluded to preserve collateral venous return from the caudal body, which was considered compromised by impaired caudal vena caval inflow.

A pump flow of approximately 60–80 mL/kg/min under hypothermic conditions was considered acceptable for a limited period of partial cardiopulmonary bypass in this case, and approximately 60 mL/kg/min was regarded intraoperatively as the minimum acceptable flow to proceed with right atriotomy. However, this was not an ideal bypass configuration for open right atrial surgery, because complete venous drainage was not achieved. The decision to proceed was based on the maintenance of acceptable partial cardiopulmonary bypass flow, the judgement that systemic perfusion was adequate based on systemic arterial pressure, serial arterial blood gas findings, and lactate concentrations, the ability to manage caudal vena caval return with suction, and the palliative goal of limited debulking to relieve life‐limiting right atrial obstruction. A strict predefined numerical abort criterion was not established, and this was considered a limitation of the procedure.

The surgical approach should also be compared with simpler previously reported techniques. Total venous inflow occlusion (TVIO) has been described for selected right‐sided cardiac procedures in dogs, including right atrial tumour resection, and may be appropriate when the lesion is surgically accessible and the anticipated intracardiac manipulation time is short (Hunt et al. 1992; Verbeke et al. 2012). TVIO was considered a potential alternative because the procedure was palliative. However, it was not selected because the mass occupied most of the right atrial lumen, was firmly adherent to the septal aspect of the right atrium, and required careful stepwise debulking rather than rapid excision. In addition, the dog had impaired venous return due to right atrial obstruction, and complete venous inflow occlusion was considered potentially poorly tolerated. The actual intracardiac manipulation time was 40 minutes, which would have exceeded the short operative window generally intended for TVIO. Therefore, partial CPB was selected to provide limited circulatory support under hypothermia and allow controlled palliative debulking.

Several important limitations should be acknowledged. Preoperative contrast‐enhanced CT, including CT angiography and thoracic CT, was not performed. Consequently, the extent of local invasion, the anatomical relationship between the mass and adjacent cardiovascular structures, and the presence of small pulmonary metastases could not be fully assessed. Although preoperative thoracic radiographs did not reveal findings suggestive of pulmonary metastasis, small pulmonary metastases could not be excluded. In addition, abdominal staging was limited to ultrasonography; therefore, complete oncological staging was not achieved. Thrombus or tumour‐associated thrombus could not be definitively excluded. Furthermore, a definitive tissue diagnosis was not obtained before surgery because preoperative sampling of the intracardiac mass was considered technically difficult and potentially hazardous. Although arterial pressure monitoring and serial arterial blood gas analyses were available during partial cardiopulmonary bypass, central venous pressure during bypass and calculated oxygen delivery indices were not recorded. In addition, caudal vena caval or hepatic venous pressure was not directly measured; therefore, the relationship between the decrease in central venous pressure and improvement in caudal venous congestion could not be definitively determined. The absence of these detailed perfusion parameters was considered a limitation of this case.

The procedure was associated with substantial perioperative and oncologic risks, including inability to establish adequate CPB, uncontrollable haemorrhage, right atrial or septal injury, air or tumour embolism, ventricular fibrillation, failure to separate from CPB, low‐output syndrome, postoperative thrombosis, incomplete tumour removal, recurrence, and possible tumour embolisation or dissemination. Ventricular fibrillation occurred approximately 10 minutes after atriotomy and required defibrillation, underscoring the potential for life‐threatening arrhythmia during intracardiac tumour manipulation under limited partial CPB.

Because the tumour protruded into the right atrial lumen and suctioned blood was returned to the extracorporeal circuit, procedure‐associated tumour‐cell recirculation or tumour embolisation could not be excluded. Although standard circuit filtration was present and no gross tumour fragments were identified in the operative field or suction reservoir, these filters cannot be assumed to remove individual tumour cells or microscopic fragments. Thus, it was not possible to determine whether the pulmonary metastasis and tumour emboli identified at necropsy represented spontaneous disease progression or were partly procedure‐associated.

The operative manipulation itself also carried substantial risk. The tumour was firmly adherent to the septal aspect of the right atrium, and necropsy later confirmed invasion into the right atrial myocardium. Aggressive complete excision could therefore have caused interatrial septal injury, right atrial wall injury, injury to major venous inflow, the coronary sinus region, or the atrioventricular junction, uncontrollable haemorrhage, or fatal arrhythmia. The residual tumour was intentionally left at the firmly adherent attachment site to relieve mechanical obstruction while avoiding catastrophic cardiac injury; if right atrial wall or septal injury had occurred, repair with pledgeted sutures or direct closure was planned when feasible, although injury involving major venous inflow, the coronary sinus region, or the atrioventricular junction might not have been salvageable.

The ethical justification for this procedure depended on thorough owner consent and recognition that it was an exceptional, high‐risk, non‐standard rescue palliative intervention. The owner was informed of the potential benefit of relieving right atrial obstruction, the lack of definitive preoperative diagnosis, the possibility of incomplete tumour removal or inadequate CPB, the risks of fatal haemorrhage, cardiac injury, tumour embolisation or dissemination, recurrence, and perioperative death, and the availability of non‐surgical or palliative alternatives.

The clinical improvement observed after surgery should be interpreted cautiously. Surgical debulking may have contributed to short‐ to medium‐term improvement by relieving mechanical obstruction of the right atrium, improving venous return, and resolving ascites. However, the oncologic effect of the procedure was incomplete and uncertain. Complete excision was not achieved, residual tumour was intentionally left in place, recurrence was documented on Day 360, and necropsy confirmed pulmonary metastasis and tumour emboli. The true onset of tumour regrowth could not be determined precisely because recurrence was likely gradual, and standardised follow‐up imaging at fixed postoperative intervals, including postoperative CT, was not performed. Therefore, the long‐term outcome cannot be attributed to surgery alone. Toceranib was initiated on Day 21, corresponding to 14 days after surgery, as postoperative medical therapy, and its potential contribution to disease stabilisation or delayed progression cannot be excluded. Radiation therapy may also have been an alternative for tumour control or disease stabilisation, although it was not expected to provide immediate relief of the intracavitary obstruction in this dog. In addition, the relatively slow‐growing natural behaviour reported for some aortic body tumours may have contributed to the observed clinical course. Thus, this case should be interpreted as showing possible temporary palliative relief of mechanical obstruction, rather than definitive evidence that surgical debulking prolonged survival or achieved meaningful oncologic control.

This approach should not be interpreted as a generally recommended treatment for dogs with heart base tumours or intracardiac tumour extension. Palliative surgery may be beneficial only under exceptional circumstances: when severe intracardiac obstruction is the major life‐limiting problem, rapid improvement cannot be expected with medical therapy, radiation therapy, or other palliative treatments alone, the intracavitary component can be debulked with acceptable technical feasibility, and advanced cardiac surgical and perfusion support are available. Careful staging whenever possible, backup cannulation strategies, and thorough owner consent remain essential.

In conclusion, partial CPB‐assisted limited debulking may provide temporary palliative relief of life‐limiting right atrial obstruction and improve clinical signs in carefully selected dogs with intracardiac extension of a heart‐base tumour. However, this approach is highly invasive, high‐risk and non‐curative, and should not be generalised as standard treatment. Its contribution to long‐term survival remains uncertain because postoperative toceranib therapy and the natural behaviour of the tumour may also have influenced the clinical course.

## Author Contributions

**Shuji Suzuki**: conceptualisation, methodology, investigation, data curation, writing.–.original draft, writing – review and editing. **Sachiyo Tanaka**: investigation, methodology, data curation, writing – review and editing. **Nobuo Kanno**: investigation, methodology, data curation, writing – review and editing. **Takuya Yogo**: methodology, writing – review and editing and supervision. **Yasuji Harada**: methodology, writing – review and editing and supervision. **Masaki Michishita**: investigation (pathology, including immunohistochemistry), formal analysis, writing – review and editing. **Yasushi Hara**: conceptualisation, supervision, methodology, writing – review and editing. All authors have read and approved the final manuscript and agree to be accountable for all aspects of the work.

## Funding

The authors have nothing to report.

## Ethics Statement

The authors confirm that the ethical policies of the journal have been adhered to. Ethical approval was not required for this case report because it describes the clinical management of a client‐owned dog performed as part of routine veterinary care.

## Consent

Written informed consent was obtained from the dog's owner for all procedures and for publication of clinical details and images.

## Conflicts of Interest

The authors declare no conflicts of interest.

## Supporting information




**Supporting File 1**: vms371244‐sup‐0001‐Video1.mp4.

**Supporting File 2**: vms371244‐sup‐0002‐Video2.mp4.

**Supporting File 3**: vms371244‐sup‐0003‐Video3.mp4.

**Supporting File 4**: vms371244‐sup‐0004‐Video4.mp4.

**Supporting File 5**: vms371244‐sup‐0005‐Video5.mp4.

**Supporting File 6**: vms371244‐sup‐0006‐SuppMat.docx.

## Data Availability

The data that support the findings of this study are available from the corresponding author upon reasonable request.
