# Supplementary material for: Palliative Debulking of an Aortic Body Tumour With Right Atrial Invasion in a Dog Under Partial Cardiopulmonary Bypass
Source: Vet Med Sci. 2026 Sep 23;12(5):e71244. doi: 10.1002/vms3.71244 (PMC13600768; doi:10.1002/vms3.71244)
Supplement: Supplementary file 1 [file VMS3-12-e71244-s003.docx]

Supplementary material

Video legends

Video 1. Echocardiographic images of the right parasternal long- and short-axis views showing an aortic body tumor with right atrial invasion.

The mass occupies most of the right atrial lumen.

Video 2. Intraoperative images during debulking of the right atrial mass.

Limited palliative debulking was performed under partial cardiopulmonary bypass; complete tumor excision was not attempted because of firm adherence to the right atrial septal aspect.

Video 3. Echocardiographic images of the right parasternal long- and short-axis views on Day 14 postoperatively.

Only minimal residual intracardiac tissue is visible.

Video 4. Echocardiographic images of the right parasternal long- and short-axis views on Day 360.

A recurrent right atrial mass is documented.

Video 5. Axial computed tomography images on Day 390.

These images demonstrate biliary obstruction.
